# Mapping the Masseteric Nerve for Facial Reanimation: An Anatomical Study of Two Dissection Strategies

**DOI:** 10.3390/medicina62010044

**Published:** 2025-12-25

**Authors:** Stefan Rössler, Wolfgang Zemann, Niels Hammer, Veronica Antipova

**Affiliations:** 1 Division of Macroscopic and Clinical Anatomy, Gottfried Schatz Research Center, Medical University of Graz, Auenbruggerplatz 25, A-8036 Graz, Austria; st.roessler@stud.medunigraz.at; 2 Department of Oral and Maxillofacial Surgery, Klagenfurt Am Wörthersee Clinic, Feschnigstraße 11, A-9020 Klagenfurt am Wörthersee, Austria; 3 Department of Oral and Maxillofacial Surgery, Medical University of Graz, Auenbruggerplatz 5, A-8036 Graz, Austria; wolfgang.zemann@medunigraz.at; 4 Department of Orthopedic and Trauma Surgery, University of Leipzig, D-04103 Leipzig, Germany; 5 Division of Biomechatronics, Fraunhofer Institute for Machine Tools and Forming Technology Dresden, D-09126 Dresden, Germany

**Keywords:** anatomical dissection, face lift, face morphology, facial reanimation, masseteric nerve, maxillofacial surgery, surgical approach, Thiel embalming

## Abstract

*Background and Objectives:* The masseteric nerve (Mn) is increasingly used for facial reanimation because of its reliable location, high axon count, low donor morbidity, and favorable clinical outcomes. Precise topographic knowledge of the Mn relative to reproducible intraoperative landmarks is essential for safe dissection. This study investigated the intramuscular position of the Mn relative to two defined reference lines. *Materials and Methods:* Seventy-two hemicrania from 36 individuals (aged 54–99 years) embalmed postmortem using the Thiel method were examined. Measurements were referenced to two defined anatomical lines: the angle–canthus line (ACL), extending from the mandibular angle to the lateral canthus of the eye, and the articular eminence line (AEL), extending from the articular eminence to the base of the zygomatic temporal process. *Results:* The Mn crossed the ACL at an average distance of 39.9 ± 5.9 mm from the mandibular angle with up to four branches. The first intramuscular branch arose 15.6 ± 4.7 mm superior to the ACL. The Mn was located 4.9 ± 1.9 mm anterior to the articular eminence and 4.7 ± 1.5 mm inferior to the AEL, coursing at an average angle of 68.5 ± 11.6° to the AEL. The AEL and ACL provide reliable and clearly defined reference lines for locating the Mn and improve intraoperative reproducibility. The Mn followed a predictable oblique course and was consistently identified in the masseter muscle (Mm) beneath an intramuscular aponeurosis. Nerve diameter varied by site, underscoring the need for standardized measurement locations. Distal localization along the ACL may enable preservation of early intramuscular branches and reduce donor morbidity. Further studies should evaluate axon counts at defined points and clarify the relationship of the Mn to the masseteric artery for better intramuscular orientation during dissection. *Conclusions:* The Mn can be located within a 63 mm^2^ area beneath the AEL at the masseter entry and more distally on the ACL. ACL-based access may protect the first intramuscular branch of the Mn and the temporal branch of the facial nerve (TBFN), and it represents a potential alternative for smile reconstruction for patients with preserved eye closure.

## 1. Introduction

The masseteric nerve (Mn) arises from the anterior division of the mandibular nerve in the infratemporal fossa as the most posterior branch and runs towards the posterior part of the mandibular notch above the lateral pterygoid muscle, anterior to the temporomandibular joint and posterior to the tendon of the temporalis muscle [1]. It is the largest motor branch of the trigeminal nerve (V) and passes over the lateral pterygoid muscle before entering the masseter muscle (Mm) [2]. Its high number of axons, its proximity to the facial nerve and the possibility of preserving the masticatory function make the Mn a popular candidate as a donor nerve in facial reanimation surgery [3,4]. Today, different there are algorithms for choosing surgical therapy in facial reanimation procedures based on the time that has elapsed since injury and on patient age [5,6]. Paralysis with a duration of up to two years can be treated with direct repair ± nerve graft, cross facial nerve graft (CFNG), masseteric and hypoglossal nerve transfers, or the Babysitter Procedure, a combination of CFNG and hypoglossal or masseteric nerve transfer [6,7,8]. Paralysis with a de-innervation time >2 years or silent electromyography requires muscle transfer due to a loss of motor end-plate function [2,5]. The use of the Mn is considered standard for unilateral paralysis in many clinics because it allows for better excursion of the oral commissure compared with CFNG innervated transplants [9]. Moreover, the utilization of Mn in operative facial reconstruction facilitates a single-stage procedure, whereas the employment of CFNG alone necessitates two surgical procedures, constituting a substantial drawback, particularly for elderly patients afflicted with comorbidities [10]. In both pediatric and adolescent patients with high nerve regeneration, CFNG with muscle transplant is the gold standard [11]. However, Mn-innervated muscle transplants should be considered for older patients >70 years with unilateral paralysis, bilateral paralysis, or Moebius Syndrome or cases involving high asymmetry or a strong contralateral smile and denial of two consecutive surgeries with CFNG [2,12,13]. In order to achieve a spontaneous smile and sufficient contraction of a muscle graft, a double-innervation procedure can be performed in one surgery using CFNG and the Mn [14]. Masseteric-nerve-innervated muscle transplants (MIMs) lead to first movement three to six months post-surgery [2,15,16]. The use of the Mn in facial reanimation is documented for paralyses after Moebius Syndrome, Bell’s Palsy, intracranial tumors, temporal bone fractures, basal skull fractures, acoustic neuroma and parotid tumors [12].

Manktelow et al. succeeded in using MIMs to reconstruct smiling with an average middle upper lip movement of 8.3 mm and an oral commissure of 13 mm; for comparison, the average maximal commissure movement of a smile in a non-paralyzed face is 14 mm [12]. Accordingly, 96% of patients were satisfied with their smile after MIMs, although only 37% reported spontaneous smiling all or most of the time [12]. Furthermore, difficulties with eating, drinking and speaking were improved, and 70% of patients did not feel disturbed by movements of the muscle graft while eating [12]. It takes more than just one muscular force vector to reconstruct a physiological smile. Boahene et al. reconstructed periorbital wrinkling in 30% with a multivector gracilis flap, which is a sign of a Duchenne smile [15]. Furthermore, they were able to increase exposure of maxillary teeth, exposure of the gingival scaffold, and interlabial exposure at the level of the canine teeth and achieved better results regarding the facial asymmetry index [15]. Possible complications of MIMs include bulkiness and the need for fat removal or secondary flap thinning, hematoma, abscesses, swelling and the need for additional tendon sling or muscle repositioning [12,13,15]. The outcome of dynamic reconstructions with the Mn is very promising. Despite promising results using the Mn as a donor nerve, there is no gold standard for access, although some methods have been described [3,17,18,19,20]. All of the methods mentioned locate the Mn in its proximal course, which carries a risk of injury to the transverse facial artery (TFA), temporal branch of the facial nerve (TBFN) and parotid gland (PG). A more distal approach could prevent bleeding, nerve damage and salivary fistula. Therefore, a better understanding of the relationship of the Mn to nearby reliable anatomical landmarks and lines appears important.

## 2. Materials and Methods

The present study was approved by the Ethics Committee of the Medical University of Graz, protocol number 35-477 ex 22/23. It was conducted at the Division of Macroscopic and Clinical Anatomy, Gottfried Schatz Research Center, Medical University of Graz (Austria) in 2025. While alive, all body donors had given their informed consent to the donation of their postmortem tissues for research purposes. All donations were bequeathed to the Division of Macroscopic and Clinical Anatomy of the Medical University of Graz under the approval of the Anatomical Donation Program of the Medical University of Graz and in accordance with Styrian legislation concerning body donations. Seventy-two facial halves obtained postmortem from 36 individuals embalmed using the Thiel original and Thiel modified methods were examined [21,22,23,24]. The age at death of the 19 male and 17 female individuals ranged from 54 to 99 years. Tissues were only included if they showed no major pathological lesions in the face region, including former surgery, or tumors in the parotideomasseteric region.

### 2.1. Dissection

**Figure 1:**
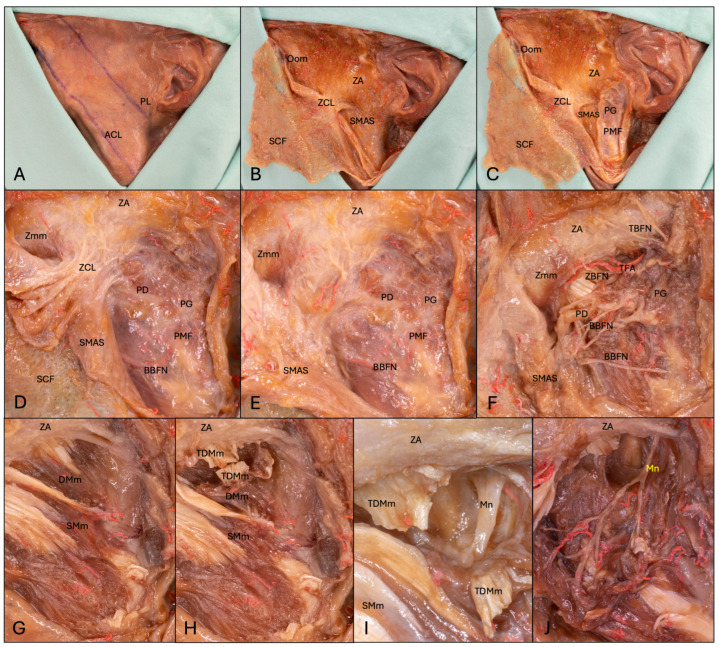
Dissection steps. (**A**) The ACL and PL were marked before dissection. (**B**) A SCF was dissected anteriorly to the Oom, ACL and ZCL via a facelift-type incision. (**C**) The SMAS overlying the PG was incised to identify the PMF and PG before going anteriorly. (**D**) A SMAS flap was created and the Zmm was identified. The PD and the buccal branch of the facial nerve (BBFN) were already through the PMF. (**E**) The ZCL was separated from the SMAS flap. (**F**) The PMF was removed and the TBFN, ZBFN, BBFN, TFA and PD were identified. (**G**) The superficial masseter muscle (SMm) and the DMm were visualized. (**H**) The TDMm was incised. (**I**) The Mn was found under the TDMm. (**J**) The intramuscular course of the Mn (highlighted in yellow) was visualized. The arterial system was injected with a radiopaque red latex mass via the common carotid artery. Abbreviations: ACL, angle–canthus line; BBFN, buccal branch of the facial nerve; DMm, deep masseter muscle; Mn, masseteric nerve; Oom, orbicularis oculi muscle; PD, parotid duct; PG, parotid gland; PL, Pitanguy’s line; PMF, parotideomasseteric fascia; SCF, subcutaneous flap; SMAS, superficial musculoaponeurotic system; SMm, superficial masseter muscle; TBFN, temporal branch of the facial nerve; TDMm, tendon of deep masseter muscle; TFA, transverse facial artery; ZA, zygomatic arch; ZBFN, zygomatic branch of the facial nerve; ZCL, zygomatic cutaneous ligament; Zmm, zygomatic major muscle.

The whole dissection and measurements were conducted with the aid of magnifying glasses (Starmed; EX 3.0- Sydney- Model A, Starmed GmbH & Co. KG, Am Schammacher Feld 19, D- 85567 Grafing, Germany), with a magnification of 3.0. First, a marking pen was used to mark the inferior border and angle of the mandible, the Pitanguy’s line (PL) and the line for the deep plane approach (Figure 1). Then, a facelift-type incision with a no. 10 scalpel was made starting 20 mm superior to the anterior edge of the ear helix at the hair line, coursing inferiorly along the hairline to the anterior edge of the helix continuing to the superior edge of the tragus. There, the incision was placed along the posterior edge of the tragus continuing in an anterior direction at the intertragic incisure following the border of the ear lobe and concha up to the post-auricular hairline. Then, subcutaneous dissection in an anterior direction to the line of the deep plane approach and lateral border of the orbicularis oculi muscle (Oom) was conducted to create a subcutaneous flap (SCF). Dissection started superior in the temporal region to facilitate the access to the subcutaneous plane and continued inferior to the mandibular angle. For better flap mobility and visibility of underlying structures, the incision was extended along the hairline at the anterior and/or posterior end of the marked incision line and horizontal to the orbital rim. Then, a pretragal incision was made down to the parotideomasseteric fascia (PMF), and dissection under the superficial musculoaponeurotic system (SMAS) was conducted to visualize the border of the PG, the facial nerve branches, the TFA and the parotid duct (PD). After identification of the TBFN and zygomatic branches of the facial nerve (ZBFN), a horizontal incision along the inferior border of the zygomatic arch (ZA) was made. Then, the zygomatic major muscle (Zmm) was visualized, and the zygomatic cutaneous ligament (ZCL) was separated from the SMAS flap and the flap was shifted anteriorly. At this point, the facial nerve branches, TFA and PD, were vaguely visible through the PMF. Following this, the fascia was removed and the aforementioned structures were relocated anteriorly. Then, the lateral pole of the temporomandibular joint, the coronoid process and the incisura semilunaris was palpated by repeated abduction and adduction of the jaw. An approximate estimation of the area where the Mn entered the Mm through the mandibular notch was made. Then, the PG was undermined and separated from the surface of the Mm to depict the deep layer of the Mm and its posterior border. The muscular dissection started with an incision 10 mm inferior to the ZA from the posterior to the anterior margin of the superficial layer of the posterior deep Mm (DMm). After separation of that muscle layer, the tendon of the middle layer of the deep Mm (TDMm) was incised and the trunk of the Mn was visualized. Then, the Mn was dissected in an antero-caudal direction. In the last five body donors, the Mn was detected with the aid of the measurements already taken over a proximal and distal approach.

### 2.2. Morphometry

Morphometrical measurements were taken with a surgical ruler, goniometer and caliper. The surgical ruler was used for measurements in relation to a line running from the mandibular angle to the lateral canthus (ACL) and a line connecting the most inferior point of the articular eminence with a point at the inferior border and base of the temporal process of the zygomatic bone (AEL). For this purpose, the muscle entry point and localization of the first intramuscular branch of the Mn in relation to the AEL, the length of the ACL, the position of the crossing point (CP) of the Mn with the ACL, and the distance of the first branch of the Mn to the ACL were investigated. The goniometer was used to measure the angle between the proximal part of the Mn to the superior border of the ZA and the ECL. The diameter of the Mn at the muscle entry point and CP with the ACL was measured with a caliper. Photos were taken during and after the dissection. IFAA recommendations for the ethical use of anatomical images were taken into account [25]. Microsoft Excel version 16.102.2 (25102623, Microsoft Corp., Armonk, NY, USA) was used to collect and analyze the collected the data.

## 3. Results

### 3.1. On the Position of the Mn Relative to the Articular Eminence and the Zygomatic Arch

The Mn was present at a distance averaging 4.9 ± 1.9 mm (2–11 mm) anterior to the articular eminence and 4.7 ± 1.5 mm (2–9 mm) inferior to the AEL. Including the above-measured values, the Mn was found at the muscle entry in a 63 mm^2^ area below the AEL. In all cases, the nerve at the muscle entry was located under an intramuscular aponeurosis. The average angle between the AEL and the proximal course of the Mn was 68.5 ± 11.6° (40–88°). The angle between the upper margin of the ZA and the Mn averaged 61.2 ± 12.0° (34–86°). The first intramuscular branch of the Mn was inferior to the EAL at an average distance of 7.9 ± 3.4 mm (3–19 mm).

### 3.2. The Relation of the Mn and Its First Branch to the Angulus-Canthus Line

**Figure 2:**
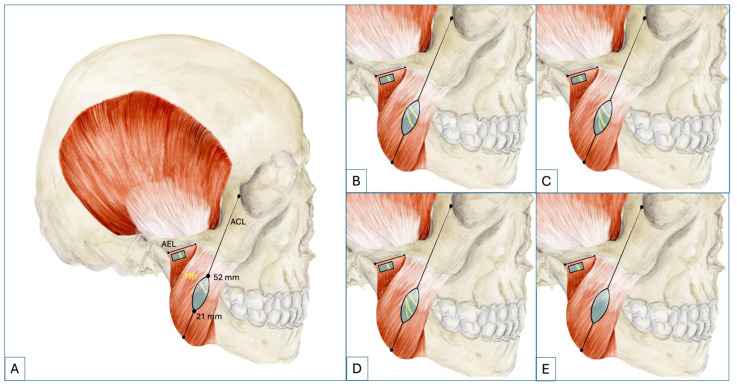
The masseteric nerve at the angle–canthus line (**A**) The Mn (highlighted in yellow) can be found at the ACL at a distance from 21 mm up to 52 mm measured from the mandibular angle. One nerve branch is present in 62.5%. (**B**) In 23.6%, two branches are present. (**C**) Three branches are present in 9.7%. (**D**) More than three nerve branches are present in 2.8%. (**E**) In 1.4%, the Mn does not cross the ACL. Abbreviations: ACL, angle–canthus line; AEL, articular eminence line; Mn, masseteric nerve.

The length of the ACL averaged 98.2 ± 7.2 mm (82–115 mm). The Mn crossed the ACL with no (1.4%), one (62.5%), two (23.6%), three (9.7%), four (1.4%) or five (1.4%) branches (Figure 2). These crossing points were located on the ACL at an average distance of 39.9 ± 5.9 mm (21–52 mm), measured from the mandibular angle. The first branch of the Mn originated superior posterior to the ACL in 100% of the cases at a distance averaging 15.6 ± 4.7 mm (4–27 mm) relative to the above-mentioned line.

### 3.3. The Diameter of the Mn at the Muscle Entry and at the ACL

The diameter of the Mn at the muscle entry averaged 1.1 ± 0.3 mm (0.6–1.7 mm) and 0.5 ± 0.2 mm (0.1–0.9 mm), respectively, at the CP with the ACL. In the 71 cases where the Mn was present at the ACL, its major branch diameter ranged between 0.2 and 0.9 mm. In 72.2% of cases, the diameter of the major branch of the Mn at the ACL was ≥0.6 mm. In cases with more than one branch at the ACL, the combined diameter of the branches ranged from 0.6 to 1.5 mm.

### 3.4. Reliability of Finding the Mn via the ACL and AEL

**Figure 3:**
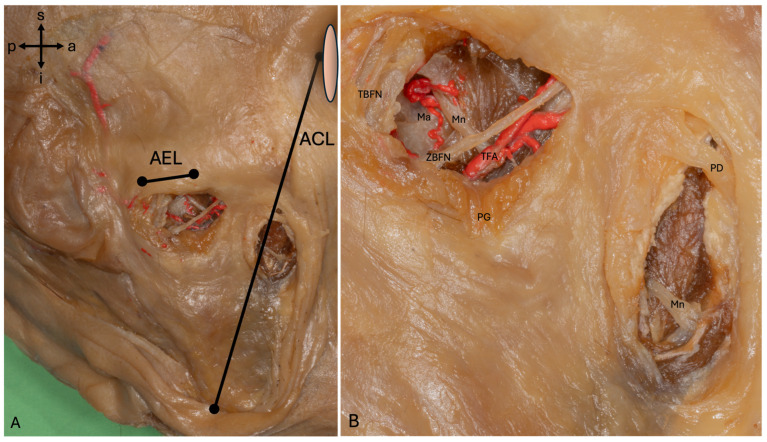
A proximal and distal approach to the masseteric nerve (**A**) This hemiface shows the proximal approach via the AEL and distal approach via the ACL. (**B**) The Mn is proximal to the zygomatic and temporal branch of the facial nerve, Ma and transverse facial artery. At the distal approach via the ACL, no arteries or nerves had to be displaced, and the parotid duct could be undermined and preserved by blunt preparation. The arterial system was injected with a radiopaque red latex mass via the common carotid artery. Abbreviations: a, anterior; ACL, angle–canthus line; AEL, articular eminence line; i, inferior; Ma, masseteric artery; Mn, masseteric nerve; p, posterior; PD, parotid duct; PG, parotid gland; s, superior; TBFN, temporal branch of the facial nerve; TFA, transverse facial artery; ZBFN, zygomatic branch of the facial nerve.

The Mn could be found in 100% of cases via the AEL and ACL with the aid of the measurements carried out (Figure 3). To access the Mn via the AEL, the TBFN and ZBFN, as well as the TFA, were retracted. 

When accessed via the ACL, the buccal branches of the facial nerve were displaced, but vessels were not present at this point, although the PD had to be undermined in two cases.

When accessing via the AEL, an intramuscular aponeurosis had to be separated, while the aponeurosis of the superficial parts of the Mm had to be split when accessing via the ACL.

## 4. Discussion

### 4.1. Reproducible Landmarks for Reliable Masseteric Nerve Localization

Surgical approaches use anatomical landmarks such as the zygomatic arch, tragus, condylar process, coronoid process, mandibular notch, frontal branch of the facial nerve and vertical lines to locate the Mn intramuscularly [3,17,18,19]. Vertical lines without a defined start and end point are highly dependent on the position of the head and are therefore only suitable to a limited extent for use in a surgical setting. With regard to the use of the frontal branch of the facial nerve, reference must be made to discrepancies with the common anatomical nomenclature, as it is unclear whether this refers to the TBFN or ZBFN. It seems obvious to utilize the ZA, condylar process, coronoid process and mandibular notch to locate the Mn, as these structures mark the area where the nerve enters the Mm. All these structures are close to the nerve and can be easily palpated intraoperatively, which facilitates orientation. In accordance with Cheng and colleagues [19], this study found abduction and adduction of the jaw helpful to better palpate the edge of the mandibular notch and also the most inferior point of the articular eminence [15]. The use of distance measurements of the Mn to predefined anatomical landmarks helps facilitate the reliable location of the Mn. Measurements from the tragus and zygomatic arch are often used, but they come with disadvantages. There is no clearly defined point at the structures mentioned from where the measurements were carried out. It is therefore difficult for inexperienced surgeons to determine the position of the Mn via these measurements without having practiced the approach by anatomical dissection. In our measurements, the starting point and direction of the measurements are clearly defined by the AEL and ACL. This facilitates the use of these measurements intraoperatively for orientation, even for less experienced surgeons. Further, a distal approach via the ACL spares structures like the PG, TFA and TBFN.

### 4.2. Angle and Path: Mapping the Proximal Masseteric Nerve

To ensure safe intramuscular preparation, it is important to know the course and depth of the Mn. Borschel and colleagues described the Mn as coursing at an angle of 50 ± 7.6° relative to the ZA, thus traversing towards the oral commissure [3]. The present study observed the proximal segment of the Mn to run at an average angle of 68.5 ± 11.6° to the AEL, and 61.2 ± 12.0° to the upper margin of the ZA, respectively. In contrast to previous measurements of this angle, this study outlined where the retractor should be placed at the ZA. According to the literature, the Mn can be found at a depth of 1.0 to 1.5 cm [18,19]. Due to the displacement of muscle tissue during preparation, no measurements were conducted with regard to depth, however, the Mn was identified under the intramuscular TDMm in 100% of cases, which is consistent with the observations of Collar et al. [18].

### 4.3. Refined Morphometry of the Masseteric Nerve at Intramuscular Landmarks

Discrepancies exist in the literature with regard to the diameter of the Mn, which can be attributed to different measuring methods and measuring points. Goh and colleagues described an average diameter of 0.8 mm at the Mm entry point [26]. Cotrufo et al. measured an average diameter of 2 mm in the proximal region of the Mn [17]. In the studies of Cheng and colleagues, the Mn nerve diameter averaged 1.63 *±* 0.63 mm, but the point of this measurement was ill-defined [19]. The given investigations measured the diameter of the Mn at clearly defined points. Here, the Mn diameter averaged 1.1 mm at the muscle entry and 0.5 mm on the ACL, respectively.

### 4.4. Beyond the Numbers: Fascicles, Branching, and the Surgical Promise of the Mn

Several studies investigated the axon number of the Mn using different measurement methods and locations, and their results differed accordingly [3,4,26]. Goh et al. described the Mn as monofascicular in 57%, bifascicular in 27% and trifascicular in 6%, respectively, consistently demonstrating one dominant fascicle with an axon count of 1395 *±* 447 axons [26]. Cotrufo et al. reported that the Mn had an average diameter of 2 mm measured with a Vernier’s caliper at its proximal part [17]. Coombs et al. examined the Mn at 460×-magnification using computer assisted planimetry, demonstrating an axon count of 1543 *±* 292 [4]. According to Borschel et al., the Mn comprises 2775 *±* 470 axons, computed by a validated and semiautomated method [3]. The present study did not determine any axon count but found the Mn to have an average diameter of 1.1 mm at its muscle entry point, and 0.5 mm at the ACL. More information on the axon number of the Mn at clearly defined localizations such as the ACL or Mm entry would be helpful for surgical planning in future.

Further studies would be helpful to explain exactly why no functional loss of the Mm occurs following the harvest of the Mn as a donor nerve. It has been stated in the literature that the Mn divides into up to 5 branches before entering the muscle [2,27]. No additional nerve branches were observed at the muscle entry point in the present study. Cotrufo et al. described one constant branch of the Mn as running in an antero-caudal direction and a second branch in 17.6% running in a vertical caudal direction after entering the Mm [17]. Abou-Al-Shaar identified two primary pedicles within the Mm using a nerve stimulator [28]. The present study observed a dominant branch, which progressively diminished in caliber along its course while after giving off multiple smaller branches. A similar observation was described by Soso, who described the course of Mn as tree-like [29]. In this study we focused on the major branch of the Mn at the ACL and its first intramuscular branch, but did not examine the consistency of different smaller branches. In our view, the function of the Mm can be partially preserved if the Mn is harvested distally to its first intramuscular branch for coaptation. This is of paramount importance in order to maintain the innervation of the coronoid part of the Mm and its stabilizing function on the temporomandibular joint to prevent potential postoperative temporomandibular joint problems [30]. According to the given measurements, this can be ensured by harvesting the Mn 19 mm below the AEL.

Joo et al. demonstrated that the Mn crosses the mandibular notch together with the masseteric artery (Ma) [1]. To prevent bleeding and usage of cauterization near to Mn during dissection and to enable indirect identification of the Mn via ultrasound, more morphometric information on the course and positional relationship of the Ma to the Mn would be helpful.

### 4.5. Limitations

The limitations of the work are possible errors in the preparation and a possible observer bias. In addition, the position of the mandibular angle also depends on the occlusion and atrophy of the jaw, which may have influenced our measurements. This study examined only the diameter and not the number of axons of the Mn on the ACL. Further, this is an anatomical study, and its results must still be tested in a clinical setting.

## 5. Conclusions

The Mn can be found both over a 63 mm^2^ area under the AEL at the muscle entry and further distally with respect to the ACL 21 to 52 mm away from the mandibular angle.

Locating the Mn via the ACL makes it possible to protect the first intramuscular branch and TBFN. Further, damage to the TFA and PG can be prevented by approaching via the ACL. In facial nerve palsy patients with preserved eye closure function and a desire to reconstruct their smiles, access via the ACL can be chosen as an alternative to conventional preparation methods. Information on the axon count of the Mn at the ACL would be useful before the use of the ACL for Mn identification in a clinical setting. Further studies would be useful to investigate the possibility of an oral approach to Mn on the ACL in order to prevent extraoral scarring.

## Data Availability

Data will be available under reasonable request to corresponding authors.

## Abbreviations

The following abbreviations are used in this manuscript:

a	anterior
ACL	Angle–canthus line
AEL	Articular eminence line
BBFN	Buccal branch of the facial nerve
CFNG	Cross facial nerve graft
CP	Crossing point
DMm	Deep masseter muscle
i	inferior
Ma	Masseteric artery
MIMs	Masseteric-nerve- innervated muscle transplants
Mm	Masseter muscle
Mn	Masseteric nerve
Oom	Orbicularis oculi muscle
p	posterior
PD	Parotid duct
PG	Parotid gland
PL	Pitanguy’s line
PMF	Parotideomasseteric fascia
s	superior
SCF	Subcutaneous flap
SMAS	Superficial musculoaponeurotic system
SMm	Superficial masseter muscle
TBFN	Temporal branch of the facial nerve
TDMm	Tendon of deep masseter muscle
TFA	Transverse facial artery
ZA	Zygomatic arch
ZBFN	Zygomatic branch of the facial nerve
ZCL	Zygomatic cutaneous ligament
Zmm	Zygomatic major muscle
